# Real-Time Observation of Magnetic Domain Structure Changes with Increasing Temperature for Z-Type Hexagonal Ferrite

**DOI:** 10.3390/ma15103646

**Published:** 2022-05-20

**Authors:** Sung-Dae Kim, Ihho Park

**Affiliations:** 1 Advanced Metals Division, Korea Institute of Materials Science, 797 Changwondaero, Changwon 51508, Korea; sdkim@kims.re.kr; 2 Department of Materials Testing & Evaluation, Korea Institute of Materials Science, 797 Changwondaero, Changwon 51508, Korea

**Keywords:** hexagonal ferrite, magnetization, magnetic moment, Lorentz TEM, in situ TEM

## Abstract

Z-type hexagonal ferrites have recently received attention for their room-temperature magnetoelectric (ME), which is activated when the temperature at which the transverse-conical spin-state transitions to a ferrimagnetic state is increased. The changes in the magnetic domain structure at the transition have been well-documented; however, they are still not understood in detail. In the present study, Lorentz transmission electron microscopy (TEM) analysis combined with an in situ heating experiment was conducted to demonstrate the shift in magnetic domain structure during the transition from the transverse-conical spin arrangement to a ferrimagnetic spin order. The dynamics of the magnetic domain structure changes with the increasing temperature were acquired in real-time. At 490 K, the magnetization transition from the transverse-conical spin state to the ferromagnetic state was demonstrated. Cross-tie domain walls formed during the magnetic transition process. The increased effect of the demagnetizing field applied to the 180° magnetic domains was caused by a lower magnetocrystalline anisotropy (MCA) at the easy axis of magnetization.

## 1. Introduction

Hexagonal ferrites (hexaferrites) are a group of ferrites with a hexagonal crystal structure. Hexaferrites have a wide range of applications, and thus, they constitute up to 50% of the total magnetic materials manufactured globally, at over 300,000 tons per year [1]. In addition to their application as permanent magnets, they are commonly used as data storage materials and components in microwave/gigahertz frequency electrical devices [2,3,4,5,6]. Using various divalent cations (Ba, Sr, and Co) and tuning the stoichiometry, hexagonal ferrites of several sorts (M, Y, Z, W, X, and U) can be produced [1,7]. Magnetoelectric (ME)/multiferroic phenomena are one of the most exciting discoveries of hexagonal ferrites [7,8,9]. Previous studies have revealed that magnetically induced ferroelectricity can be manifested by complex internal arrangements of magnetic moments in hexagonal ferrites, in which ferromagnetic properties are intrinsically coupled to their atomic structures [1,7,8,10,11,12,13,14]. Recently, Z-type hexagonal ferrites have been highlighted from the viewpoint of their high-temperature and low-field ME operations [7,8,11]. Z-type hexagonal ferrites consist of a stack of tetrahedral and octahedral Fe-Co layers along the c axis. It is known that the X-type hexagonal ferrites have a collinear ferrimagnetic ordering below 670 K, where the net magnetic spin moments are arranged in a parallel manner by alternating large (L) and small (S) blocks along the c axis. Preserving the transverse-conical spin-state in a wide temperature range is key to realizing the room-temperature ME effect in a low magnetic field from Z-type hexaferrite. The improved ME property of Z-type hexagonal ferrites originates from the transition temperature elevation [7,8,10,11,13,14], where the transverse-conical spin state transforms into a ferrimagnetic state. Although the magnetic domain structure changes at the transition have previously been reported, it has not yet been well understood. In this study, we investigated the temperature-dependent magnetic domain structure of a Z-type hexagonal ferrite using a combination of Lorentz transmission electron microscopy (TEM) and in situ heating TEM techniques, which enables the observation of a magnetic domain structure change at an elevated temperature.

## 2. Materials and Methods

Z-type hexaferrite single crystals (Ba_0.5_Sr_2.5_Co_2_Fe_24_O_41_) were grown from a Na_2_O-Fe_2_O_3_ flux. The chemicals for the hexaferrite (BaCO_3_, SrCO_3_, CoO, Fe_2_O_3_, and Na_2_O) were melted at 1420 °C in a Pt crucible, followed by heat treatment at 900 °C under O_2_ flow for 8 days to remove oxygen vacancies [10]. The final product was confirmed to be a single phase using X-ray diffraction (XRD). XRD analysis was conducted at room temperature using CuK_α_ radiation at 40 kV and 30 mA (X’pert Pro PANalytical diffractometer, Malvern Panalytical Ltd., Malvern, United Kingdom). Needle-shaped (0.3 mm × 3 mm × 0.3 mm) single-crystal hexaferrite samples were used to measure the magnetic properties. The temperature and magnetic field were controlled using a physical property measurement system (PPMS^TM^, quantum design, San Diago, CA, USA). In all measurements, a magnetic field was applied along the $\left[11\bar{2}0\right]$ direction. TEM samples were prepared by mechanical polishing followed by Ar^+^ ion milling (E. A. Fischione 1020, E.A. Fischione, Export, PA, USA) with a liquid nitrogen-cooled cold stage. Cross-sectional samples were observed using a TEM (JEM 2010-Lab_6_, JEOL Ltd., Tokyo, Japan) with an acceleration voltage of 200 kV. Lorentz TEM images were acquired under magnetic-field-free conditions, through the free-lens control function in the TEM. An in situ TEM heating stage (652 double-tilt heating holder, Gatan Inc., Pleasanton, CA, USA) was used to heat the sample. A heating rate of 100 °C/min was used to reach the target temperature. Magnetic domain structures were reconstructed using the transport of intensity equation (TIE). The TIE method [15,16,17] was applied using the QPt plugin (HREM Research Inc., Tokyo, Japan) on the Digital Micrograph software (Gatan Inc., Pleasanton, CA, USA). Sets of bright-field (BF) TEM images with under/over (defocus value (Δf) = ±192 mm) and in-focus were used as input data for the TIE analysis.

## 3. Results and Discussion

**Figure 1:**
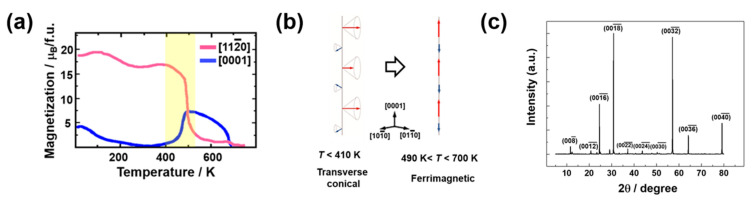
(**a**) Magnetization vs. temperature curves for $\left[11\bar{2}0\right]$ and [0001] directions measured at *μ*_0_*H* = 50 mT (**b**) Schematic of the transition of the magnetic spin state (**c**). XRD pattern of the single phase Z-type hexagonal ferrite.

Figure 1a shows the magnetization of the Z-type hexagonal ferrite as a function of temperature measured at 50 mT. The magnetization drops along the $\left[11\bar{2}0\right]$ direction are observed at approximately 410, 490, and 680 K. Meanwhile, the magnetization parallel to the [0001] direction (c-axis) increases at 410 K followed by drops at 490 and 680 K. This suggests that the hexagonal crystal’s transverse-conical spin state on the basal plane is stabilized below 410 K. Furthermore, the easy axis of the magnetization changes parallel to the *c*-axis at 490 K, followed by the paramagnetic state above 680 K. Similar magnetization transitions were observed in the magnetic properties measurement using PPMS on Ba_3_Co_2_Fe_24_O_41_ hexaferrite [8]. The transition of the magnetic spin state is illustrated in Figure 1b. The magnetic symmetry of a cycloidal spiral spin arrangement is known to induce spontaneous ferroelectric polarization where the magnetic propagation vector ($\vec{q}$) lies in the spiral plane. The origin of magnetically driven ferroelectricity can be successfully explained by the spin current and inverse Dzyaloshinskii–Moriya models [18,19]. These models can also be applied to the transverse-conical spin arrangement in Z-type hexagonal ferrites. XRD pattern of the sample is shown in Figure 1c. The diffraction peak intensity maxima is observed for around 2θ = 30.80, corresponding to a plane of $\left(00\bar{18}\right)$. This c-plane should be appeared dominantly when the specimen of the hexagonal structure is ground down, and to favorable orient normal to the c-plane for the XRD measurement. The peaks were well indexed to the Z-type hexaferrite, with a space group P63/mmc and lattice parameters a = 5.87 (3) Å and c = 52.23 (2) Å (ICSD #98328), which suggests that the majority phase of the sample is Z-type hexaferrite.

**Figure 2:**
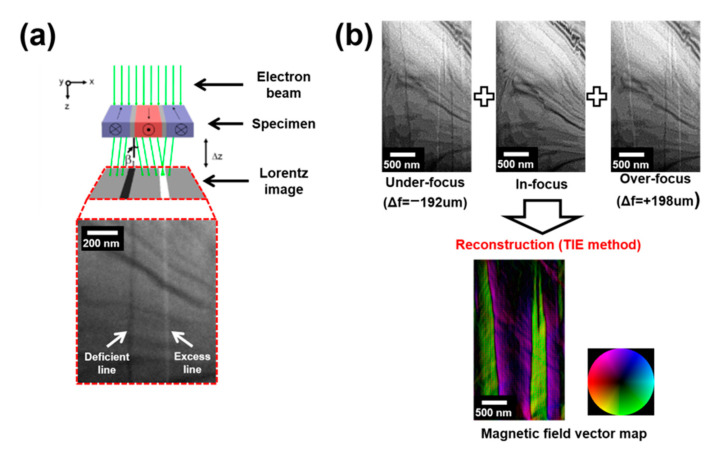
(**a**) Schematic of a ray diagram showing the paths of electrons passing through a magnetic specimen (upper), together with the magnetic domain or domain wall contrast for the Fresnel modes (lower). (**b**) The schematic diagram for imaging the in-plane magnetic configurations using Lorentz TEM. Three images were acquired in the same region under three different defocus conditions. The in-plane magnetization distribution map could be generated from the TIE method. The color wheel represents the magnetization direction at every point.

Lorentz TEM experiments were performed to visualize the magnetic domain structure change during the transition from the transverse-conical spin arrangement to the ferrimagnetic spin order [20,21]. Lorentz TEM uses interactions between the electron beam and magnetic fields formed by the local magnetization distribution in the crystals [15,16,17,20,22]. One of the most common techniques for Lorentz TEM imaging is the Fresnel mode (Figure 2a), where the internal magnetic field on the TEM sample deflects the electron beam that is deficient (electrons are deflected away from the domain wall) or excess lines (increased electron density caused by the converged electrons) in the image plane. The intensity inversion of the magnetic contrast at the domain walls can be seen between the over- and under-focused images because the appearance of the deficient and excess lines is dependent on the focusing condition (Δf). The intensity inversion at the magnetic domain wall was used to reconstruct a magnetization vector map. The TIE can be used to visualize the magnetic field distribution in a magnetic sample using sets of defocused Lorentz TEM (Fresnel) images [15,16,17]. Mathematically, the TIE corresponds exactly to the Schrödinger equation for high-energy electrons in a vacuum under the small-angle approximation [15]. This equation relates the phase distribution to the intensity distribution and its derivative, relative to the direction of wave propagation [15,16,17]. Figure 2b demonstrates the reconstruction of the magnetization vector map in the Z-type hexagonal ferrite by applying the TIE method to a set of in/de-focused TEM images.

**Figure 3:**
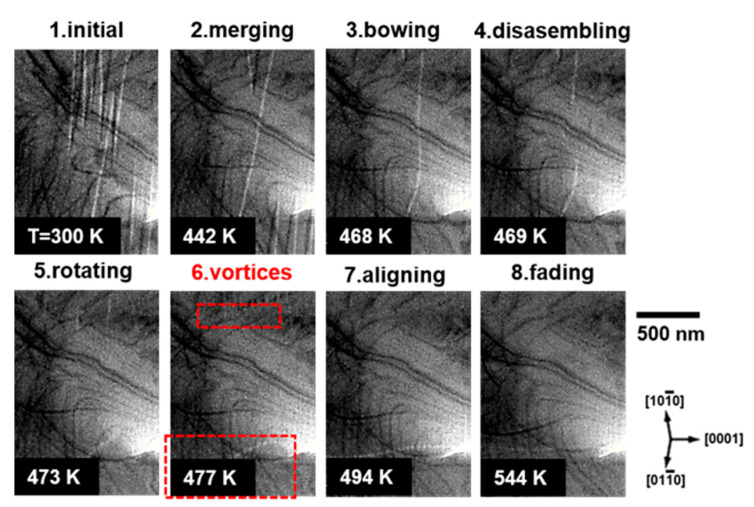
Sequential BF-TEM images of the Z-type hexagonal ferrite via in situ heating.

The temperature-dependent dynamics of the magnetic domain evolution of Z-type hexagonal ferrite are shown in Figure 3 and Supplementary Video S1. Spike-shaped in-plane antiparallel magnetic domains were stabilized at room temperature (300 K). As the temperature increased, the magnetic domains merged. At approximately 460 K, the magnetic domain walls parallel to the basal plane were bowed and instantly disconnected at 469 K. Subsequently, the direction of the disconnected domain walls was rotated parallel to the c-axis at 477 K. Above 500 K, the c-axis-aligned domain walls became faint and finally faded away, indicating that the sample was in a paramagnetic state.

**Figure 4:**
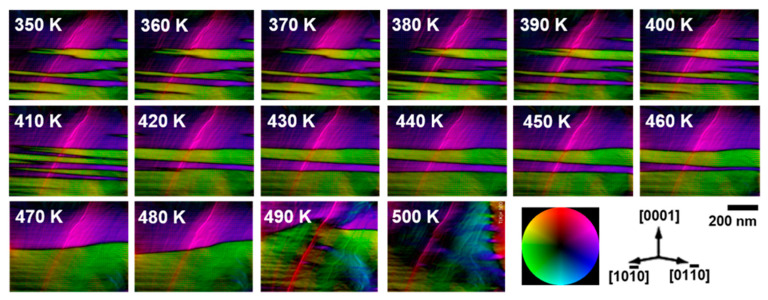
Sequential images of the magnetic vector maps of the Z-type hexagonal ferrite by heating from 350 to 500 K. The color wheel represents the magnetization direction at every point.

Figure 4 illustrates the temperature-dependent magnetic domain structure of the Z-type hexagonal ferrite. A magnetization map was produced using the TIE method. The green and purple regions in Figure 4 correspond to in-plane antiparallel magnetic domains. A stack of reconstructed magnetization map images is provided in Supplementary Video S2. Below 500 K, the magnetic domain walls were parallel to the basal plane of the crystal. Upon increasing the temperature from 300 K, the green magnetic domains grew laterally on the basal plane and subsequently became thicker by coarsening with neighboring parallel magnetic domains. At 490 K, the magnetic domain structure became simple, with some in-plane antiparallel magnetic domains. As the temperature reached 500 K, the direction of the magnetic domain was completely rotated in the ab-plane normal direction (parallel to the c-axis).

**Figure 5:**
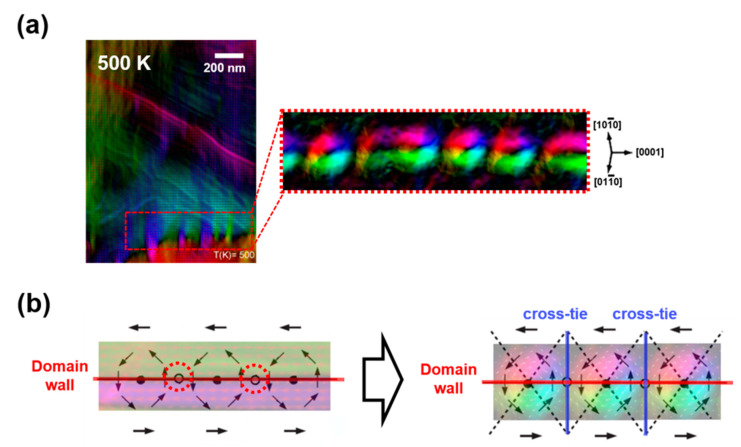
(**a**) The cross-tie domain wall evolved after the transition of the magnetic spin axis, (**b**) schematic diagram of 180° magnetic domain wall structure flux closures within the planes without (left) and with (right) the presence of the cross-tie walls.

A noticeable point in the magnetic transition process is the appearance of the cross-tie domain wall right after the rotation of the magnetic domain wall (Figure 5a). The energy per unit area of a conventional domain wall is expressed as σw∝(AKe)12, where $K_{e}$ is the effective anisotropy constant and *A* is the exchange constant [22]. At the magnetic transition temperature, the constants associated with magnetic anisotropy were minimized, yielding a weak magnetocrystalline anisotropy (MCA) of the sample [1,7,22]. Under low anisotropies, demagnetizing fields that are normal to the domain wall can rotate the magnetization in each domain in the direction of the wall to make the flux closure represented by the red-dotted circles in Figure 5b (left) [23]. However, this flux closure is only possible in alternate intervals, and the magnetostatic energy density is increased between these regions. The flux flow discontinuity in the circles can be accommodated by developing the cross-tie walls shown in Figure 5b (right) [23,24,25]. The cross-tie walls reduce the angular shift in the flux direction normal to the main wall. This configuration gives rise to such periodic magnetic vortices across the main 180° domain wall, as shown in Figure 5a [26].

## 4. Conclusions

The temperature-dependent changes in the magnetic domain structure of Z-type hexaferrite single crystals (Ba_0.5_Sr_2.5_Co_2_Fe_24_O_41_) were investigated. The dynamics of the magnetic domain structure changes with the increasing temperature were acquired in real-time. The magnetization transition from the transverse-conical spin state to the ferromagnetic state at 490 K was demonstrated by the combined use of Lorentz TEM and in situ heating techniques. During the magnetic transition process, cross-tie domain walls were developed. The reduced MCA at the easy axis of magnetization resulted in an increase in the effect of the demagnetizing field applied to the 180° magnetic domains.

## Data Availability

Not applicable.

## Supplementary Materials

The following supporting information can be downloaded at: https://www.mdpi.com/article/10.3390/ma15103646/s1, Video S1; Video S2.
